# Molecular Mechanisms of Natural Carotenoid-based Pigmentation of Queen Loach, *Botia dario* (Hamilton, 1822) Under Captive Condition

**DOI:** 10.1038/s41598-019-48982-9

**Published:** 2019-08-29

**Authors:** Partha Sarathi Tripathy, Ningthoujam Chaoba Devi, Janmejay Parhi, Himanshu Priyadarshi, Arun Bhai Patel, Pramod Kumar Pandey, Sagar Chandra Mandal

**Affiliations:** College of Fisheries, Central Agricultural University (Imphal), Lembucherra, Pin- 799210 Tripura India

**Keywords:** Genetics, Molecular biology

## Abstract

The genetic basis and expression patterns of key genes are important aspects of study to understand the colouration. This trait differs between wild and domesticated fish which is a matter of research. *Botia dario* is an indigenous fish, having ornamental and aesthetic value, which shows faded appearance in terms of colour in domesticated condition than wild. In the present study the carotenoid-fed *B*. *dario* were examined through incorporation of marigold petal meal in the diets at the rate of 5, 10 and 15% w/w along with wild fish. The carotenoid content of tissues that is skin, muscle and intestine along with intensity of colouration increased in a dose dependant manner of carotenoid in the diet. Important carotenoid-based colouration genes that is *csf1r*, *BCDO2*, *SR-B1*, *MLN64*, *STAR5*, *GSTA2* and *PLIN2* were characterized in the fish, to find out their role in fish pigmentation. The significant difference (p < 0.05) in the expression of these genes in different tissues, when compared among carotenoid-fed domesticated and wild fish, revealed the mechanism responsible for faded colouration and also revealed the means to enhance colour in the fish.

## Introduction

The pigment patterns in vertebrates are principally studied to understand cellular and molecular mechanism of pattern development^1–3^. In case of birds and mammals, pigment patterns are mostly based on melanocyte breakdown and the transfer of melanin to different organs^4,5^. But in case of fish and amphibians, colouration is due to various classes of chromatophores (pigment cells) that is xanthophores, melanophores, iridophores, and so on which are retained intracellularly^4^. These chromatophores are responsible for the development of pigment patterns for a variety of functions among fish species^6–8^.

Recent years have seen remarkable signs of progress in our understanding of the developmental genetics with regard to patterning of embryonic axes, tissues and organ rudiments. But very little is known about mechanisms underlying the expression of traits throughout developmental stages, especially in later stages and also in adults^9^. Identifying the genes and cell behaviours underlying trait expression is a vital step in understanding the genesis of naturally occurring trait variation and evolution of form^10,11^. One ecologically essential trait is the externally observable pigment pattern of ornamental fish.

In captive culture system, fish have no access to carotenoids and therefore, the required carotenoids must be added to the diet^12^. The loss of pigments can be overcome by the addition of carotenoids to the artificial diet. Carotenoids are the primary source of pigmentation in the skin of fish^13^. Carotenoids, supplied by marigold petal meal as a supplement in the diets,s also play an important role in regulation of skin and muscle colour in fish^14,15^. Out of total carotenoid, marigold flower contains 92.5% of lutein which is a yellow-orange pigment^16^. For this reason, in the present study, this flower meal has been used as a supplement in the experimental diets.

*Botia dario* belongs to family Cobitidae of order Cypriniformes and found in Brahmaputra and Ganges basins of Bangladesh, Bhutan and Northeast India^17^. The captive breeding of this species has been studied in earlier researches^18,19^. This species has a great demand in ornamental fish trade in many countries due to its unique band pattern and bright colour^20^. It can be used as a model for studying both xanthophore and melanophore pigmentation because of its black and yellow band patterns. An early study on birds^21^ listed eleven candidate genes, responsible for different aspects of carotenoid metabolism, whereas in fish a little has been studied regarding this important mechanism. In the present study, seven candidate genes for carotenoid-based pigmentation that is *csf1r*, *BCDO2*, *SR-B1*, *MLN64*, *STAR5*, *GSTA2* and *PLIN2* were characterized in *B*. *dario* and their expression patterns were studied, using qPCR both in natural as well as experimental conditions by feeding carotenoid-rich diet.

## Results

### RNA isolation

Total RNA, isolated from various tissues, was tested for its quantity, purity and integrity. The concentration of the isolated RNA sample was found in the range of 725.65–1300 ng µl^−1^. A260: A280 ratio of the isolated RNA sample was recorded in the range of 1.9–2.0. Integrity of the extracted RNA was verified by running it on a 2% agarose gel.

### Gene sequence analysis

The partial nucleotide sequences were 671 bp, 534 bp, 274 bp, 349 bp, 806 bp, 411 bp and 466 bp for *csf1r*, *BCDO2*, *GSTA2*, *MLN64*, PLIN, *StAR5* and *SR-B1*, respectively for the genes. In the BLAST result, *csf1r* and PLIN showed the highest similarity with *Danio rerio*. The *SR-B1*, *BCDO2* and *MLN64* showed the highest similarity with *Sinocyclocheilus grahami*. Gene *StAR5* showed the highest similarity with *Cyprinus carpio* and *GSTA2* with that of *Hypopthalmichthys nobilis* in the BLAST result. GC content of gene sequences of *csf1r*, *SR-B1*, *StAR5*, *GSTA2*, *PLIN*, *MLN64* and *BCDO2* was found to be 50.84, 51.15, 52.83, 43.90, 49.14, 49.28 and 45.89%, respectively.

### qPCR analysis

**Figure 1:**
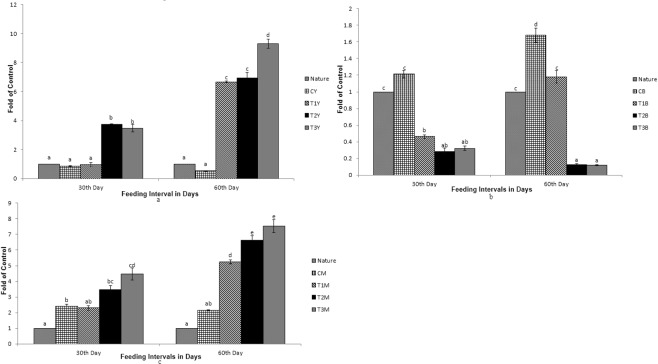
Expression pattern of *csf1r* gene; (**a**) Expression pattern in yellow skin bands with respect to Nature, where CY, T1Y, T2Y, T3Y are yellow skin tissues of control, T1, T2, and T3 respectively. (**b**) Expression pattern in black skin bands with respect to Nature, where CB, T1B, T2B, T3B are black skin tissues of control, T1, T2, and T3 respectively. (**c**) Pattern in muscle with respect to Nature, where CM, T1M, T2M, T3M are muscle tissues of control, T1, T2, and T3 respectively. Bars represent means ± s.d. Different letters over bars indicate significant difference (P < 0.05).

**Figure 2:**
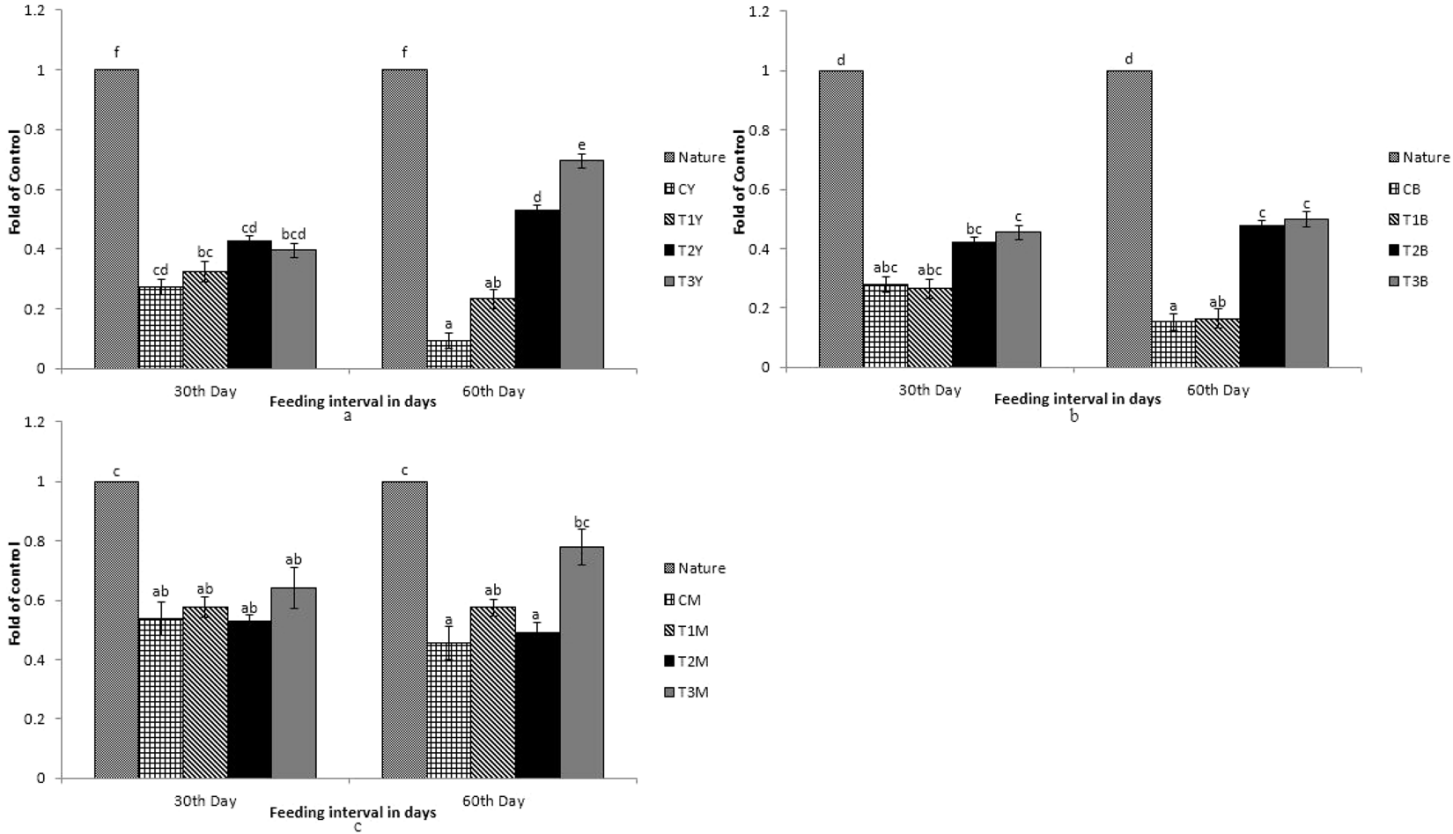
Expression pattern of *BCDO2* gene; (**a**) Expression pattern in yellow skin bands with respect to Nature, where CY, T1Y, T2Y, T3Y are yellow skin tissues of control, T1, T2, and T3 respectively. (**b**) Expression pattern in black skin bands with respect to Nature, where CB, T1B, T2B, T3B are black skin tissues of control, T1, T2, and T3 respectively. (**c**) Pattern in muscle with respect to Nature, where CM, T1M, T2M, T3M are muscle tissues of control, T1, T2, and T3 respectively. Bars represent means ± s.d. Different letters over bars indicate significant difference (P < 0.05).

**Figure 3:**
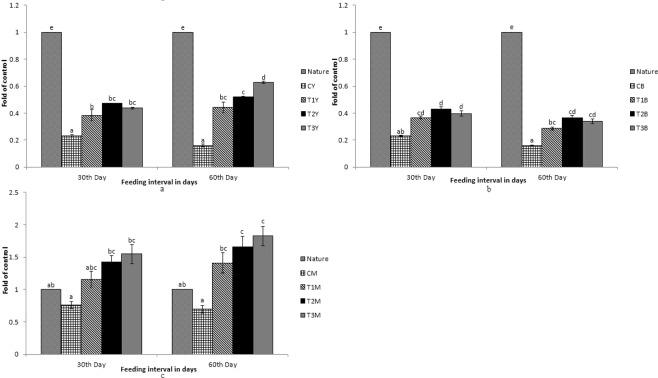
Expression pattern of *GSTA2* gene; (**a**) Expression pattern in yellow skin bands with respect to Nature, where CY, T1Y, T2Y, T3Y are yellow skin tissues of control, T1, T2, and T3 respectively. (**b**) Expression pattern in black skin bands with respect to Nature, where CB, T1B, T2B, T3B are black skin tissues of control, T1, T2, and T3 respectively. (**c**) Pattern in muscle with respect to Nature, where CM, T1M, T2M, T3M are muscle tissues of control, T1, T2, and T3 respectively. Bars represent means ± s.d. Different letters over bars indicate significant difference (P < 0.05).

**Figure 4:**
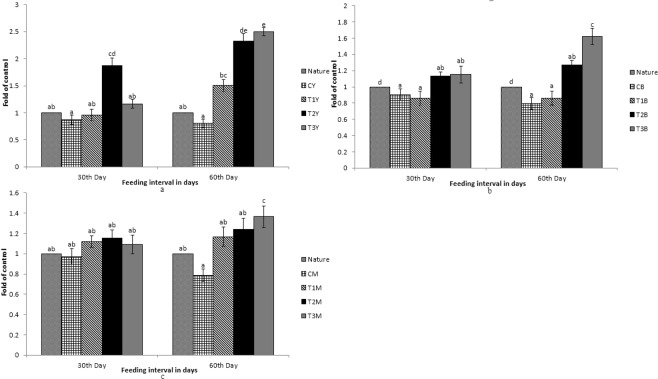
Expression pattern of *MLN64* gene; (**a**) Expression pattern in yellow skin bands with respect to Nature, where CY, T1Y, T2Y, T3Y are yellow skin tissues of control, T1, T2, and T3 respectively. (**b**) Expression pattern in black skin bands with respect to Nature, where CB, T1B, T2B, T3B are black skin tissues of control, T1, T2, and T3 respectively. (**c**) Pattern in muscle with respect to Nature, where CM, T1M, T2M, T3M are muscle tissues of control, T1, T2, and T3 respectively. Bars represent means ± s.d. Different letters over bars indicate significant difference (P < 0.05).

**Figure 5:**
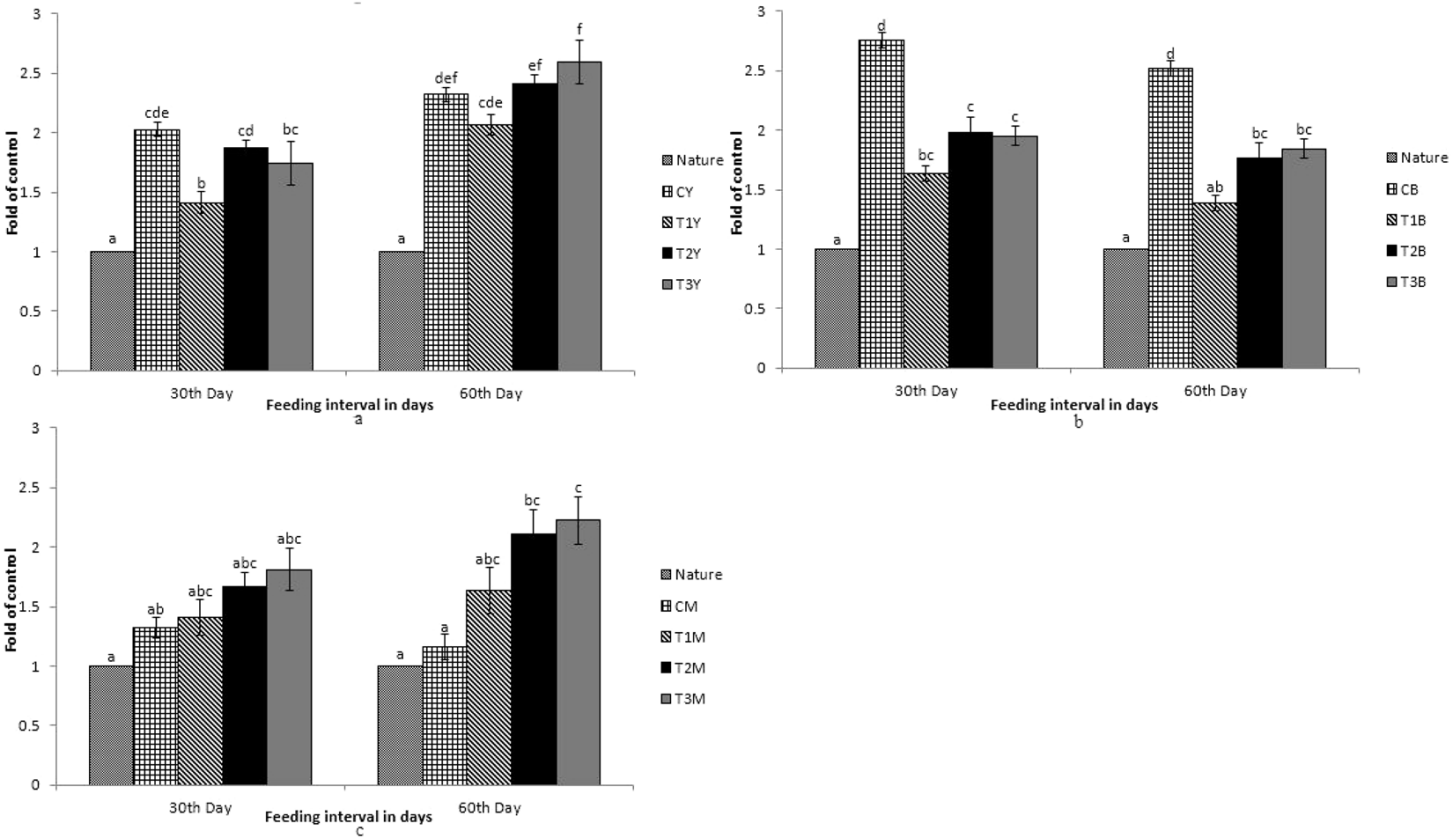
Expression pattern of *PLIN* gene; (**a**) Expression pattern in yellow skin bands with respect to Nature, where CY, T1Y, T2Y, T3Y are yellow skin tissues of control, T1, T2, and T3 respectively. (**b**) Expression pattern in black skin bands with respect to Nature, where CB, T1B, T2B, T3B are black skin tissues of control, T1, T2, and T3 respectively. (**c**) Pattern in muscle with respect to Nature, where CM, T1M, T2M, T3M are muscle tissues of control, T1, T2, and T3 respectively. Bars represent means ± s.d. Different letters over bars indicate significant difference (P < 0.05).

**Figure 6:**
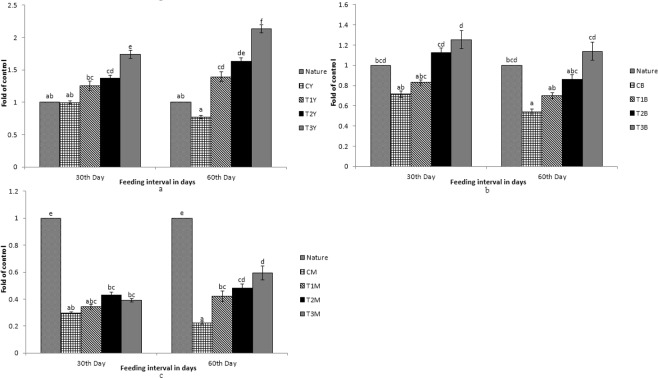
Expression pattern of *StAR5* gene; (**a**) Expression pattern in yellow skin bands with respect to Nature, where CY, T1Y, T2Y, T3Y are yellow skin tissues of control, T1, T2, and T3 respectively. (**b**) Expression pattern in black skin bands with respect to Nature, where CB, T1B, T2B, T3B are black skin tissues of control, T1, T2, and T3 respectively. (**c**) Pattern in muscle with respect to Nature, where CM, T1M, T2M, T3M are muscle tissues of control, T1, T2, and T3 respectively. Bars represent means ± s.d. Different letters over bars indicate significant difference (P < 0.05).

**Figure 7:**
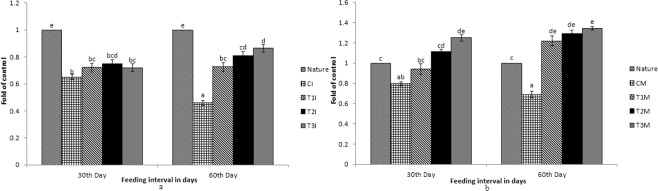
Expression pattern of *SR-B1* gene; (**a**) Expression pattern in intestine with respect to Nature, where CI, T1I, T2I, T3I are intestine tissues of control, T1, T2, and T3 respectively. (**b**) Expression pattern in muscle with respect to Nature, where CM, T1M, T2M, T3M are muscle tissues of control, T1, T2, and T3 respectively. Different letters over bars indicate significant difference (P < 0.05).

As shown in Figs 1–7, differential expression was observed in the carotenoid-based genes that is *csf1r*, *BCDO2*, *GSTA2*, *MLN64*, *PLIN*, *StAR5* and *SR-B1* due to dietary inclusion of carotenoid through marigold petal meal. Expression pattern of the genes increased significantly (p < 0.05) as compared to the control group. Expression pattern in T3 diet fed fish was the highest for all the genes in different tissues as compared to the control group. Expression pattern of the genes in nature that is fish sampled immediately from the river was compared with the expression pattern of fish from the experiment.

While comparing between nature and control group, the expression pattern of *BCDO2*, *GSTA2*, *MLN64* and *StAR5* were found to be high in nature with regard to yellow skin band, black skin band and muscle. But the *csf1r* was expressed more in yellow skin of the fish, collected from nature than the control group. The *SR-B1* was expressed more in the intestine and muscle of the fish, collected from nature as compared to the control group. Expression of *BCDO2*, *GSTA2*, *MLN64* and *StAR5* showed higher expression in yellow skin, muscle and black skin in T3 than other treatments. But the expression of *csf1r* and *PLIN* gene was higher in black skin of the control group.

While comparing the experimental fish with nature, it was found that the *csf1r*, *MLN64*, *PLIN* and *StAR5* gene showed higher expression in yellow skin band of T3. Whereas the expression of *csf1r*, *BCDO2* and *GSTA2* showed higher expression in the black skin band of fish, collected from nature. *StAR5* and *BCDO2* showed higher expression in the muscles of the fish collected from nature.

The expression of *SR-B1* in intestinal tissue was found to be high in the fish, collected from nature as compared to the control. When the control group of fish were compared with the treatments, the expression was found to be high in T3. But the expression remained low as compared to the fish, collected from nature. Interestingly, significant expression of *SR-B1* was found in muscles and intestine only and no significant expression of other genes was found in the intestine.

### Carotenoid content of tissues

Carotenoid content of the tissues that is yellow skin, black skin and muscles were calculated by the Olson method^22^ and results are shown in Table 1. Carotenoid content of the yellow skin, black skin and muscles were found to be the highest in T3 group fish on 60^th^ day and in nature as well. Carotenoid contents, measured in different tissues, are shown in Table 1.

**Table 1:** Carotenoid content of tissues in different days of feeding.

Sample	Nature	C (µg/100 mg)	T1 (µg/100 mg)	T2 (µg/100 mg)	T3 (µg/100 mg)
Yellow kin	296.23 ± 0.04°	103.24 ± 0.03^g^	151.53 ± 0.04^k^	157.56 ± 0.06^l^	166.39 ± 0.04^m^
Black skin	212.14 ± 0.01^n^	93.24 ± 0.10^f^	112.32 ± 0.03^i^	107.96 ± 0.09^h^	122.47 ± 0.05^j^
Muscle	45.76 ± 0.09^e^	6.32 ± 0.04^a^	8.87 ± 0.05^b^	9.90 ± 0.04^c^	12.67 ± 0.04^d^

Carotenoid content of different tissues at 60^th^ day of feeding fish compared with nature in means ± s.d. Different letters in superscript indicate significant difference (*p* < 0.05).

### Colorimeter analysis

**Figure 8:**
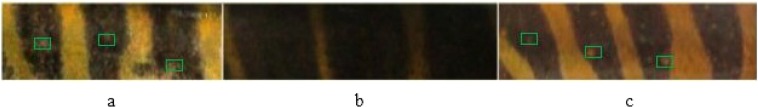
(**a**) Skin of *B*. *dario* of Nature, (**b**) skin of 60^th^ day *B*. *dario* of control group, (**c**) skin of 60^th^ day *B*. *dario* of T3 group. Formation of yellow spots over black bands are shown in green boxes.

The natural and experimental fish tissues were taken for colorimeter (Hunter Lab Scan XE, USA) analysis. The calculations for yellowness and redness were done by the NFI method^23^. Whole fish was taken for colorimeter analysis and results are shown in Table 2. Yellow patches were observed over black skin bands in Nature (Fig. 8a). Interestingly, such yellow patches were also observed on 60^th^ day in control and T3 group fish as shown in Fig. 8b,c, respectively. But, these yellow patches were higher in T3 group of fish than that of control.

**Table 2:** Hunter Lab Colorimeter analysis.

Days	Yellowness				Redness			
Nature	37.11 ± 0.54				11.49 ± 0.22			
Experimental Groups	C	T1	T2	T3	C	T1	T2	T3
15^th^ Day	12.90 ± 0.07^c^	13.26 ± 0.02^d^	17.29 ± 0.05^f^	19.84 ± 0.05^g^	0.39 ± 0.01^b^	2.40 ± 0.02^d^	4.33 ± 0.02^g^	4.84 ± 0.02^h^
30^th^ Day	12.94 ± 0.04^c^	16.68 ± 0.03^e^	20.01 ± 0.02^h^	22.62 ± 0.03^j^	0.37 ± 0.01^b^	3.49 ± 0.02^e^	5.30 ± 0.02^i^	5.42 ± 0.06^j^
45^th^ Day	11.78 ± 0.03^b^	17.70 ± 0.04 ^f^	23.32 ± 0.01^k^	27.00 ± 0.01^l^	0.93 ± 0.01^c^	3.81 ± 0.06^f^	6.11 ± 0.02^k^	7.52 ± 0.03^m^
60^th^ Day	8.43 ± 0.04^a^	20.59 ± 0.02^i^	27.47 ± 0.02^m^	30.70 ± 0.02^n^	0.10 ± 0.01^a^	3.83 ± 0.02^f^	7.29 ± 0.01^l^	7.99 ± 0.01^n^

Hunter Lab Colorimeter analysis. Intensity of colouration of different days of feeding and nature fish in mean ± s.d. Different letters in superscript indicate significant difference (*p* < 0.05).

### Sensory evaluation test

The yellow patches were found to be more in T3 group, whereas the black bands were found to be highest in control group. Based on the 1–9 point hedonic scale the panellists stated that the T3 group fish showed highest overall colouration. The significant differences for different parameters observed by the panellists are shown in Table 3.

**Table 3:** Sensory evaluation test.

Parameters	Rating by panellist on 1–9 point hedonic scale			
Experimental groups	C	T1	T2	T3
Intensity of yellow	4.5 ± 0.02^b^	4.7 ± 0.07^b^	6.0 ± 0.02^e^	7.2 ± 0.02^g^
Intensity of black	5.2 ± 0.03^cd^	5.1 ± 0.05^cd^	4.5 ± 0.07^b^	3.6 ± 0.03^a^
Overall colouration of fish	4.3 ± 0.02^b^	4.7 ± 0.07^b^	6.2 ± 0.07^f^	6.7 ± 0.05^f^
Fin pattern	5.3 ± 0.07^d^	5.6 ± 0.03^de^	5.4 ± 0.02^cd^	5.4 ± 0.06^cd^
Standard body shape	5.5 ± 0.02^cd^	5.3 ± 0.02^c^	5.2 ± 0.08^cd^	5.2 ± 0.04^cd^
Overall health status	5.4 ± 0.01^cd^	5.3 ± 0.04^cd^	5.5 ± 0.06^cd^	5.5 ± 0.05^cd^

Sensory evaluation test. Ratings of 60^th^ day experimental fish expressed in mean ± s.d. Different letters in superscript indicate significant difference (*p* < 0.05).

## Discussion

The colony stimulating factor 1 receptor (*csf1r*) is a type III receptor tyrosine kinase gene, important for xanthophore pigment development^24,25^. Carotenoids are stored in xanthophore cells which are located mainly in the skin and *csf1r* gene is involved in the migration of these cells for pattern formation^26^. Its expression was up-regulated with increase in number of feeding days and carotenoid content of the diets. The faint appearance of yellow colouration was found over black skin (Fig. 8), suggesting that *csf1r* has a role in migration of xanthophores. This finding is in conformity of the earlier work done by researchers^27^.

Carotenoids, after breakdown, are stored in muscles and integuments. β, β-carotene-9′,10′-dioxygenase 2 (*BCDO2*) gene encodes for a carotenoid-cleavage enzyme and shows a substrate specificity en route for a variety of dietary carotenoids and catalyses, an oxidative cleavage at position C9′, C10′ ^28^. Up-regulation of *BCDO2* in skin was due to increase in carotenoid uptake. However, it was not at par with nature, probably due to the source of carotenoid. The *BCDO2* has carotenoid scavenging activity for which it catabolizes the carotenoids into xanthophylls^28,29^. The finding supports the earlier works on the role of *BCDO2* gene^28–30^.

Metastatic Lymph Node Gene 64 Protein (*MLN64*) or StAR-Related Lipid Transfer Domain Containing 3 (*StAR3*), Perilipin 2 (*PLIN*) and StAR Related Domain Containing 5 (*StAR5*) genes are responsible for carotenoid binding and deposition as studied in birds^21^. Earlier study on primates concludes the role of *MLN64* in having high affinity towards lutein-binding^31^ and the marigold meal supplemented as a carotenoid source, in the present study, contains about 80% lutein^16^. Therefore, up-regulation of *MLN64* gene in skin of the experimental fish might be due to the carotenoid binding and deposition, most probably lutein. Up-regulation of *MLN64* and *STAR5* in both yellow and black skin with an increase in the number of feeding days and carotenoid level in the diets suggests that these genes are primarily involved in carotenoid deposition in the skin. In earlier studies, it has been found that *StAR5* gene is responsible for transport of lipids and cholesterol^32^. *PLIN* gene is also associated with lipid binding and lipid droplet formation which helps in storage of neutral lipids^33^. Up-regulation of *PLIN* gene was only seen in yellow skin and its down-regulation in black skin might be due to the fact that only some xanthophores migrated to the black skin leading to less deposition of xanthophores. Glutathione-S-Transferase Alpha 2 (*GSTA2*) is a key gene for carotenoid-binding protein^21,34^. Although *GSTA2* is also involved in the deposition of carotenoids, it was down-regulated in the treatment group of fish as compared to the fish collected from nature. The expression increased in yellow skin and decreased in black skin with increase in the number of feeding days. This might be due to high carotenoid binding activities in yellow skin than black skin.

Muscles are mainly the site for deposition of carotenoids from where pigment is redistributed to integuments^35^. The genes which are primarily involved in the carotenoid binding and deposition that is *MLN64*, *GSTA2* and *PLIN* were up-regulated in muscles of the treatment group of fish than nature. Expression of these genes increased significantly with increase in number of days of feeding and it was found to be the highest in the fish of T3 group. *csf1r* is involved in the migration of xanthophore cells^26^ and up-regulation of this gene in muscles in a time dependant manner of feeding clearly states that this gene plays a very important role in redistribution or migration of xanthophore cells from muscles to skin.

The main function of *StAR5* gene is the transfer of lipids and lipoproteins. Since carotenoids are hydrophobic compounds, they can’t move freely through cell plasma. Hence, they are transported to the adjacent tissues with the help of lipoproteins, especially high-density lipoproteins^36^. Expression of *StAR5* gene was up-regulated in muscles with increase in days of feeding and carotenoid level in the diets. But the main function of this gene is lipid transfer of which carotenoid distribution is just a part. Since lipid-rich feed is higher in natural condition and its transfer is guided by several other genes, it might be the reason for down-regulation of this gene in the treatment group of fish as compared to nature. Uptake of carotenoid primarily takes place in the intestine^36^ and *SR-B1* is the key gene involved in this mechanism^21^. Its expression in the intestine was much higher. But it also showed expression in the muscles which was up-regulated with increase in number of feeding days and carotenoid level in the diets, indicating some uptake of carotenoid in the muscles too.

Carotenoids, obtained from the feed, can’t be easily solubilized in the aqueous environment as they are hydrophobic in nature and their digestion, absorption and transportation are associated with lipids. Carotenoid uptake happens primarily in the proximal intestine. *SR-B1* is the key gene, involved in the uptake of carotenoid. Increase in carotenoid in feed showed up-regulation of *SR-B1* gene, especially in the intestinal tissues. The absorption of carotenoids in the intestine is influenced by esterified or non-esterified carotenoids^36^. *SR-B1* gene supports diffusion of pigments like lutein and carotenoid into the intestinal membrane^37^. It has been studied earlier that *SR-B1* gene is involved in lipid metabolism^38^. Carotenoid absorption is associated with lipids, clearly influencing the uptake of carotenoids in intestine. In another study, *SR-B1* knock-out model of mice showed lower intestinal absorption of carotenoids^39^.

Fish are incapable of synthesizing carotenoids in their own body and hence, they obtain carotenoids from dietary sources such as plants and algae. In nature, carotenoids are abundantly available for fish as they can directly obtain it from aquatic plants. This may be the reason for better colouration of fish in nature than the domesticated ones. *BCDO2*, responsible for carotenoid breakdown, and *GSTA2*, responsible for carotenoid deposition, were up-regulated in nature than in the treatment. Fish feed randomly in nature and carotenoid uptake in different fish is in different form like lutein, astaxanthin, zeaxanthin and so on. In nature, the breakdown of carotenoid is a major process in fish for the uptake of the correct form of carotenoid. This may be the reason for which *BCDO2* was highly expressed in nature both in skin and muscles than the treatment, as the treatment group of fish were fed with diets fortified with specific carotenoid source. The enrichment of feed with various sources of carotenoids may enhance colouration. The study on *GSTA2* shows that it plays a major role in the detoxification of environmental toxins and products of oxidative stress^40^. In nature, fish are subjected to many unknown environmental toxins and stress. This may be the reason why the expression of *GSTA2* was higher in nature. The expression of *csf1r*, *MLN64*, *PLIN* and *StAR5* genes in the skin was less in nature as compared to the treatment. With an increase in the amount of carotenoids in diets T1 to T3, up-regulated *csf1r*, *MLN64* and *PLIN* genes expression were noticed in both skin and muscle. Since the uptake of carotenoids primarily takes place in the intestine, *SR-B1* gene is up-regulated in the intestine in nature. But dietary inclusion of carotenoids increased *SR-B1* gene expression in muscles, indicating its possible role in deposition of these pigments.

Significant increase in the carotenoid level was found in all the tissues with increasing days of feeding and carotenoid level in the diets (Table 1), suggesting absorption of carotenoid due to dietary uptake. Carotenoid content of muscle clearly showed that absorption of carotenoid in muscle was slower than that of skin in case of domesticated fish as compared to nature. Intensity of yellowness and redness of whole fish increased with increasing days of feeding and carotenoid level in the diets but was low as compared to nature (Table 2). It suggests that longer period of feeding with carotenoid-rich diets enhances pigmentation and to maintain it, continuous inclusion of carotenoids is required in the diet. The present study will lead to future studies regarding inclusion of a combination of carotenoids in the diets from different sources and its effect on colouration. Moreover, this study will also help in understanding the role and accumulation of carotenoids in skin and muscle as well as determination of carotenoid metabolism pathway in fish.

## Methods

### Ethical statement

All the fish of *B*. *dario*, used in this study, were collected from Maharani Dam, Gomati River, Tripura, India. Ethical approval, specimen collection and maintenance were performed in strict accordance with the recommendations of the ethical standards of the Institute of Animal Ethics Committee, College of Fisheries, Central Agricultural University, Imphal, India.

### Experimental diets

Four iso-nitrogenous diets *viz*. control (C), treatment 1 (T1), treatment 2 (T2), treatment 3 (T3) of 35% crude protein were formulated by using locally available feed ingredients including rice bran, mustard oil cake, fish meal, soybean meal, corn flour and marigold petal meal and the details of feed formulation is given in Table 4. The control (C) was prepared without fortification of marigold petal meal. Other diets such as T1, T2 and T3 were formulated by fortification of 5, 10 and 15 g of marigold petal meal per 100 g feed, respectively. The dosages of carotenoids through marigold petal meal were decided after studying the effects of dietary carotenoid levels in fish^41–44^. The marigold petals were initially dried at 37 °C to prevent loss of any carotenoids in the resultant petal meal.

**Table 4:** Feed formulation of the experimental diets (% dry matter).

Ingredients	C	T1	T2	T3
Fish meal^a^	27	27	27	27
Soyabean^b^	26	26	26.5	26.5
MOC^a^	10.5	10.5	10.5	10.5
Broken wheat^a^	25	21.5	15	12
Corn starch^a^	5	3.5	4.5	2.5
Marigold^c^	0	5	10	15
Vit-min premix^d^	2	2	2	2
Sunflower oil^e^	4	4	4	4
BHT^f^	0.1	0.1	0.1	0.1
Vit-C^g^	0.2	0.2	0.2	0.2
CMC^h^	0.2	0.2	0.2	0.2

^a^Obtained from feed mill of College of Fisheries, Lembucherra, Tripura, India.

^b^Manufactured by Sister company, Agartala, Tripura, India.

^c^Bought from the local flower market, Agartala, Tripura, India.

^d^Vit-Min premixes manufactured by Anand International Biopharma, New Delhi.

^e^Saffola sunflower oil, Mumbai.

^f^Butylated Hydroxy Toluene (BHT) manufactured by Merck Limited, Mumbai.

^g^Manufactured and marketed by Himalaya Herbal Health Care, Mumbai.

^h^Carboxymethylcellulose (CMC), manufactured by Himedia Laboratories Pvt. Ltd., Mumbai.

### Experimental set-up

Live *B*. *dario*, with an average weight of 3.0 ± 0.08 g, were acclimatized for 14 days in aquarium with the control diet. Fish were maintained in a 100 L capacity 2.5 × 1 × 1.5 ft glass aquarium with aerated and filtered dechlorinated water. After acclimation, fish were randomly divided into 4 groups (3 tanks per group, 15 fish per tank). The tanks or fish groups were named as C, T1, T2 and T3 with their replicates according to the fortification of the experimental diets. The tanks were kept at an experimental condition (temperature: 26 ± 2 °C, pH: 7.1 ± 0.3, ammonia: less than 0.3 mg/L and dissolved oxygen: 6.41 ± 0.31 mg/L). The fish were fed two times (08:00 and 18:00 h) a day for a period of 60 days at 3% of body weight. The tanks were provided with aquarium stones. Thirty per cent of total water was exchanged every day from the bottom of the tanks.

### Tissue collection

The samples were collected for analysis from freshly collected riverine fish that is nature and at two different feeding days of the experiment that is 30^th^ and 60^th^ day. For the purpose of tissue collection fish were anesthetized by using clove oil (50 µl in 1 litre of water). Tissues like yellow skin, black skin, intestine and muscle were collected and preserved in TRIzol for RNA isolation and kept at −80 °C until further use.

### RNA isolation and cDNA synthesis

Total RNA was isolated from the yellow skin, black skin, intestine and muscle of *B*. *dario* by TRIzol method^45^. The quality and quantity of isolated RNA was checked with 2% agarose gel and Biospectrometer (Eppendorf, Germany), respectively. cDNA synthesis was done from the isolated RNA samples, using iScript™ cDNA Synthesis Kit (Bio-Rad, USA) according to the manufacturer’s protocol. The concentration of RNA, used for cDNA synthesis for PCR amplification and qPCR, was 1 µg/µl. All the cDNA samples were PCR amplified with a house-keeping gene that is *18S rRNA* and checked with 1.5% agarose gel for confirmation.

### PCR amplification of target genes

#### Primer designing for PCR

For characterization of target genes in *B*. *dario*, primers were designed from the conserved area of reported sequences of the genes from closely related species through NCBI-primer blast^46^. The primers, used in this study for PCR amplification of different genes, is shown in Table 5.

**Table 5:** List of primers used in both PCR and qPCR analysis.

Sl. No.	Gene Name	Forward Primer	Reverse Primer	Product Length
**PCR Primers**				
1	csf1r	TGGTGRTCACGGAGTACTGTY	ATGTGCTGGAACCTSGAMSC	671
2	SR-B1	TGATCAAYGGMACAGCSGGGG	GTTTGMAGAGAGCGGCTACA	466
3	StAR5	ATTACATAMASACKGCCAGATCK	AGAGGCTTCAAYCAYCCRTG	411
4	GSTA2	AGCARGTGCCTTTGGTKGAAA	GAGCCGTGCTGAYGTTCAYY	274
5	PLIN2	TGAAGTCTGTSTGYGAGGTGG	CTTCAGASCACATGTGTGGT	806
6	StAR3/MLN64	CGTYCGAYCTKCTYTTCATCA	GAGAGTGTATCTGGCMGCAG	349
7	BCDO2	GCTTTGATGCAYCGCTTTCA	AGTGTTCATYGAGCAGCCCA	534
**qPCR Primers**				
1	csf1r	GAGTGGCCAAAATCTGCGAC	GGCATTCTGCTGTGGGAGAT	175
2	SR-B1	GTCGGGTCTTCTGAACGTGG	GACATACACCCGGAGACAGG	162
3	StAR5	GAGTTCGCAGGATGCGTCTA	CAGAACCGTCACCCCTTCAG	144
4	GSTA2	AGCTTGTGCAGACAAAGGCTA	ACCACCTGACAACAAGCAGAA	166
5	PLIN2	AACGTTCACCTCGGTTGCTT	TGCTAAAGAGGCAGTGACAGG	177
6	StAR3/MLN64	GTTTTGGCCGTGTTCAGGTT	TACGTGCTTCCCATCACCTC	176
7	BCDO2	TGACGTACCGGAGTCGTTTTT	TTCAGCGTTTCCTGACCAGA	137

#### Amplification of partial nucleotide sequence

For partial nucleotide sequence, PCR reaction was performed with the designed primers by adding 12.5 µl of 2X master mix (Thermo Scientific, USA), forward and reverse gene-specific primers 1 µl each (10 pmol/µl), 1 µl of template cDNA (50–100 ng/µl) and nuclease-free water to make it up to a total volume of 25 µl. The PCR condition used was an initial denaturation at 94 °C for 5 minutes, final denaturation at 94 °C for 1 minute, annealing (55 °C for *csf1r*, 58 °C for *BCDO2*, 52 °C for *GSTA2*, 52 °C for *PLIN2*, 54 °C for *SR-B1*, 54 °C for *MLN64* and 56 °C for *STAR5*) for 1 minute, initial elongation at 72 °C for 1 minute, final elongation at 72 °C for 7 minute, followed by hold at 4 °C. The PCR products were checked using 1.5% agarose gel for confirmation.

#### Sequencing of PCR products

The PCR products were purified by GeneJET Gel Extraction Kit (Thermo Scientific, USA), using the manufacturer’s protocol. The purified PCR products were sequenced, using PCR primers of respective genes (Table 5) in ABI 3500 Genetic Analyzer (Applied Biosystems, USA) according to the manufacturer’s protocol. The sequences were matched in the NCBI database, using the BLAST algorithm^47^ for confirmation.

### qPCR for gene expression study

#### Primer designing for qPCR

For gene expression analysis of target genes (*csf1r*, *BCDO2*, *SR-B1*, *MLN64*, *STAR5*, *GSTA2* and *PLIN2*) in *B*. *dario*, primers were designed from the partial nucleotide sequence of different genes, using NCBI-primer blast^46^. All the primers were first checked with stored cDNA samples for confirmation. The primers, used for Real-time PCR study of different genes, is shown in Table 5.

#### qPCR analysis

StepOnePlus™ Real-Time PCR (Applied Biosystems, USA) based on SYBR green chemistry was used to study the expression analysis of different genes, using gene-specific primers designed from its sequence amplified across the collected cDNA samples. Real-time PCR reaction was performed by adding 5 µl of SYBR green master mix (Thermo Scientific, USA), forward and reverse gene-specific primers 1 µl each (1 pmol/µl), 1 µl of template cDNA (1 µg/µl) and nuclease-free water to make it up to a total volume of 10 µl. As an internal control, housekeeping gene that is *18S rRNA* was utilized for expression level normalization. Three technical replicates were made for each sample during Real-time PCR. The analysis was done by Pfaffl method^48^.

### Estimation of carotenoid content of *B. dario* fish tissues

Total carotenoids were determined by following the Olson method^46^. At the end of the feeding trial, the yellow skin, black skin and muscles of the experimental fish were analyzed to get total carotenoids content in it. 0.1 g of sample was weighed quickly and placed in a 5 ml screw-capped tube. To this 0.25 g of anhydrous sodium sulfate was added and the sample was gently smashed with a glass rod against the side of the vial to mix with anhydrous sodium sulfate. The vial was sealed after adding 0.5 ml of chloroform and placed at 0 °C overnight. The chloroform formed a clear layer above the residue. The optical density of the layer was read at 380, 410, 440, 450, 460, 475 and 500 nm wavelength in a BioSpectrometer (Eppendorf, Germany), taking 0.3 ml aliquots diluted to a column of 3 ml of absolute ethanol. A blank prepared in a similar manner was used for comparison. The wavelength at which maximum absorption obtained was used for calculation, using the formula: Total carotenoid = (Absorption at maximum wavelength × 10)/(0.25 × amount of sample taken in g).

### Determination of colour by the colorimeter

**Figure 9:**
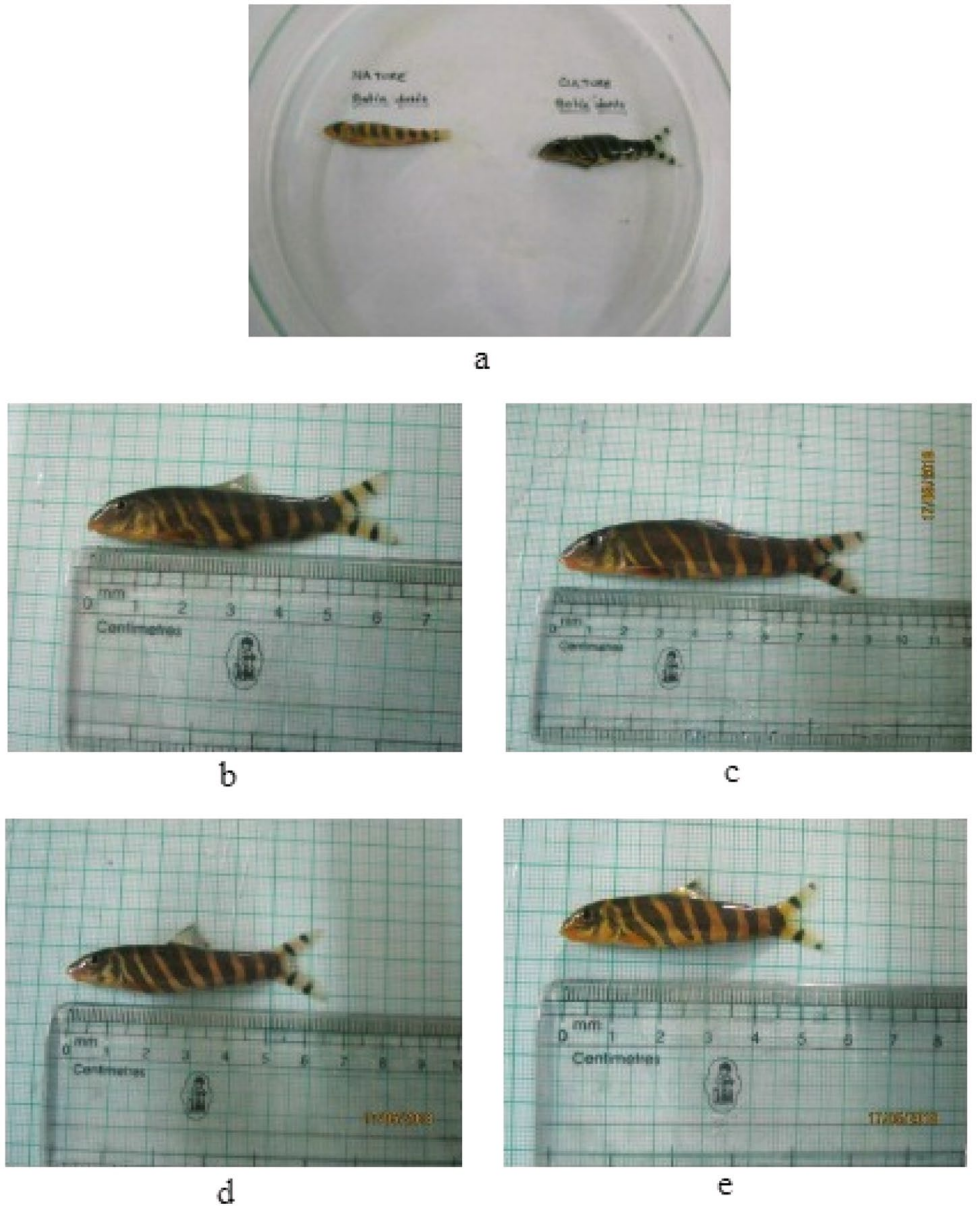
Experimental fish at different feeding days; (**a**) Comparison between Nature and domesticated fish, (**b**) 60^th^ day control group fish, (**c**) 60^th^ day T1 group fish, (**d**) 60^th^ day T2 group fish, (**e**) 60^th^ day T3 group fish.

The colour measurement was made by using a Colorimeter (Hunter Lab Scan XE, USA) according to the manufacturer’s instruction. The tristimulus L*a*b* measurement mode was used as it relates to the human eye response to colour. The L* scale represents lightness (L* = 0 for black, L* = 100 for white), the a* scale represents the red/green. (+a* intensity in red and −a* intensity in green) and the b* scale represents the yellow/blue (+b* intensity in yellow and −b* intensity in blue). The redness and yellowness were calculated using the following equation^23^: Redness = Cos(16°) a* + Sin(16°) b* and Yellowness = −0.7[−Sin(16°) a* + Cos(16°) b*]. The fish of different treatment groups and control (60^th^ day of the experiment) along with Nature are shown in Fig. 9. The photographs of Nature versus domesticated, 60^th^ day control and 60^th^ day T1, T2 and T3 group fish was taken and are shown in Fig. 9a–e, respectively.

### Sensory evaluation test

Test panels of persons were randomly recruited to judge colour. The treatments were not revealed to the individuals who were asked to rank the fish according to the intensity of colour and other health parameters. The rankings were scored on a scale of 1–9 point hedonic scale (one being the lowest) for each treatment groups.

### Statistical analysis

The carotenoid content of fish tissues, gene expression, colorimeter analysis and sensory evaluation tests are presented as mean ± standard deviation. All the data were analyzed by one-way analysis of variance (ANOVA), using SPSS v16.0. When ANOVA identified differences among groups; multiple comparisons among means were made with Duncan’s new multiple range tests. Statistical significance was determined by setting the aggregate type I error at 5% (P < 0.05) for each set of comparisons.

## Data Availability

The sequences are submitted to NCBI database with accession numbers MH384789, MK214361, MK214362, MK214363, MK214364, MK214365 and MK214366 for *csf1r*, *SR-B1*, *StAR5*, *GSTA2*, *PLIN*, *MLN64* and *BCDO2*, respectively.
